# Regeneration of Nerves

**Published:** 1869-05-01

**Authors:** Alphonse Lavaren, Ch. Robin


### TRANSLATIONS.

### *Regeneration of Nerves.*

BY ALPHONSE LAVAREN AND CH. ROBIN.

[Translated for the Journal from Schmidt's Jahrbucher, Leipsig, Jan., 1869.]

Alphonse Lavaren, by means of experiments upon pigeons and young rabbits (Recherches experimentales sur la regeneration des nerfs. Téhse de Strasbourg, 1868. 4.) has arrived at the following conclusions respecting the regeneration of nerves:

1st. Nerves divided or resected at short intervals, can be reunited by a permanent cicatrix of nerve substance.

2d. The nutrition of nerve depends upon the influence of nutritive centers, which are principally emitted with nerve ganglia.

3d. If a nerve be separated from its nutritive center, it becomes atrophied.

4th. The degeneration of a divided nerve is thus determined, in that the nerve tissue undergoes fatty transformation and is absorbed.

5th. The degeneration seizes, simultaneously, the entire peripheral section of a divided nerve.

6th. The reunion of the two segments of a divided nerve is brought about through a tissue, which is rich in young cells.

7th. It seems to follow, that of this tissue, the blood-clot, between the two segments of the nerve, organizes itself.

8th. In the midst of these cells the nerve-fibre originates, in the same manner as the nerve-fibre in the embryo developes itself.

9th. The regeneration can not take place if the two segments of the divided nerve are widely separated, or if they lie in an old, suppurating wound.

10th. The repair, then, begins in the peripheral nerve-segments, if the connection with the nutritive centers is restored.

11th. The essential element of reparation lies in this: that the nerve-fibre, a secretion from the nuclei of the intervening cell-membrane, becomes again visible.

12th. The reparation seems to proceed from the center to the periphery.

Upon the publication of Lavaren's valuable work, Charles Robin (Journ. de l'Anat, et de la Phys. v. 3. p 321. Mai et Juin, 1868) has followed with a brief statement of his own observation of the nerve-reparation above-mentioned. The regeneration begins with the formation of a rich connective tissue, of a reddish, grayish-red, or yellowish-red tint, between the primitive bundles of the two segments, which gradually, in the interstices between, proceeds to where previously the resected nerve lay. In this is found only embryo-plastic substance (the connective-tissue, and young cells of Lavaren and others), together with an amorphous substance and capillaries. At first this tissue is weak, and easily torn, and also rich in vessels. Robin particularly urges the existence of a fine, amorphous, granulated mass between the nuclei and capillaries. The nuclei are partly ovoidal; the ovoidal exist in so much larger numbers, that very speedily the section is filled up by them.

After a couple of days a central connective-tissue of ovoidal nuclei is formed, and appears as fibro-plastic, spindle-form, or stellate cells (plastic cells of other authors). From this the amorphous substance is formed, and of it the entire tissue seems to consist. In this tissue Robin discovered, in dogs and rabbits, from sixteen to twenty-five days after the division of the nerve, for the first time, nerve-fibre, or nerve-tube, which resembled those in the embryo.

The nerve-tubes are formed of elongated, ovoidal nuclei.

A number of nuclei combine in the formation of the single nerve-tube. These nuclei are distinguished from the embryo-plastic nuclei, in that they are longer, narrower, paler, and more finely granulated. They lie in the same direction, in ranks one behind another, yet without touching; the narrow interstices between are filled with a pale, finely granulated mass, which is as wide as the nucleus, and so they appear as thin bands, with parallel edges, which seem at first to be actually in contact. These bands are, at first, 0.003 to 0.004 Mm. in width, becoming gradually wider, yet increasing in length, while the nuclei push out toward one another, and, indeed, at first somewhat more rapidly. These bands are 0.005 to 0.007 Mm. in width, about eight to ten weeks after the nerve division has taken place, and resemble Remak's bands; and now they undergo a succession of changes, from which it is evident that they correspond to the nerve-tube. Just as the embryonic development of the peripheral nerves is formed, so also, here, the external membrane of the nerve-tube, and not the axis-cylinder, is the first part of the central nerve-tube which appears. That is to say, the axis-cylinder is not, here, the first part of the nerve-tube which appears.

These bands approach each other simultaneously, and in great numbers. In the middle group of bundles, which is not easily separated from the surrounding connective-tissue, begin the principal changes in the nerve-tubes, and, further off, the same appear in the peripheral bundle; the fine granulation disappears, the bands become paler, and in the center, more transparent, and, upon the sides, appear two parallel pale lines of 0.001 Mm. in thickness.

At this stage of the regeneration the gray or Remak's band is enclosed in nerve-tube. This increases rapidly in size, the nuclei remain enclosed in the membrane, and the granulations lose themselves more and more in the membrane. In consequence of this development the number of nuclei diminish, and they themselves become smaller.

In the canal of the tubular membrane is observed a homogeneous, whitish, strongly refracting fluid, which is recognized as fibre, or myeline. From the ninth week, and often as early even as the sixth week, the myeline may be recognized in the form of globules, or accumulated in isolated spots of the membrane; whereby these acquire a varicose appearance.

An axis-cylinder is first determined in three or four weeks after the section of the nerve. S. C. B.

